# Famous Poisoners in Fiction

**Published:** 1893-08-26

**Authors:** 


### Famous Poisoners in Fiction.

### III.—CATHERINE.

A story with a purpose, such is Thackeray's immortal Catherine. He had an aim in view—to write of things as they were, to illustrate life faithfully, and thus to counteract the evil wrought by those once-popular romances which, to quote his own words, "created interest in their rascals by making them perform virtuous actions." He succeeded in drawing a character sketch, the more loathsome, the more awful, because instinctively the reader knows, feels, that such women have existed in all the ages past, and may, can, *do* exist in the time present.

And yet so subtle is the genius of the author that withal a feeling of sympathy arises in the heart of at least every woman reader for the sinful, betrayed, vulgar, selfish woman pourtrayed in the few short pages which contain the "Story of Catherine." In spite of all she is a woman, no demon of nether earth if no angel of light, but a woman, even though a bad woman, for she loved, although she loved unworthily, and hated as fiercely as only a woman can hate. Her unpremeditated confession of her premeditated guilt alone proves her true womanhood. Would ever devil, would ever man, confess in a moment of overpowering repentance a deeply-planned sin? No, none but a woman, a frail woman, the prey of her feelings, swayed to and fro, as a pendulum, by the impulses of her wild, untamed nature, and variable as the shifting weather-cock, turned hither and thither by the whirling wind, would thus betray herself. Poor Catherine, as well as betrayed, disappointed, revengeful, cruel Catherine, thy fellow-women must ever feel a pang of pity as well as a shudder of horror as they read the history of thy fate! thy sin!

But saddest of all is the thought that there are now living prototypes of this bad woman. Their love turned to hate, despising themselves, abhorring mankind, just because one man, the man they loved, has failed them, these miserable as well as sinful women walk this earth which God once pronounced to be very good, but which man has rendered vile indeed. With hearts filled with anger and bitterness, with the sting of that sorrow which leadeth not to repentance in their tortured souls, they wander hither and thither, administering the mental poison of mistrust, of cankering loathing for things pure, bright, beautiful to those fellow-men and fellow-women with whom they come in contact on their way through life. Alas! in most cases the poison is gladly, willingly taken, for if the influence for good of holy men upon their fellow-men is marvellous, more terribly marvellous is the awful power of the influence of evil, and especially when this fearful weapon rests in the hands of a beautiful woman.

The story is one of low life, a story of the people., differing thus from Bulwer Lytton's Lucretia. Although a Child surely of the Night of sin, of cruelty, of hatred, of death, Catherine knows little, nothing, of the fine art of poisoning. She does not argue; she does not study; she does not with shifty step tread softly the long corridors; she simply, with determined hate, acts abruptly—openly. A visit to several chemists with the common transparent plea of a bad tooth; a punchbowl prepared by her own hands; these are the murderess's weapons; with these tools she seeks her revenge—and fails at the last moment when she has virtually succeeded because that, in her woman's heart she still preserves for her wicked betrayer that strange, perverse, overpowering passion which men call love! Insulted, degraded, tortured, distorted, a dream thing of beauty no longer, but not dead, not dead for she is a woman—and with women love triumphs over everything. Not even betrayal, desertion, cruelty, curses, blows, scorn, can quench its burning flame—nay, oftentimes it seems as if they added but fuel to its fierce fire.

Laudanum! It is a dangerous weapon, this tool of the poor woman's spite; would that it were a hundred times less easy to obtain than it is. Maybe there is many a Catherine to-day who has not saved herself by confession, who has committed the foul deed of murder, who has beheld her victim laid quietly in the grave, and into whose heart remorse, born too late, is eating into the very flesh, causing an unavailing sorrow which can be healed never more.

Had not Catherine possessed the curse of beauty she would not have been tempted, and would not have fallen. A beautiful girl from the Poor House. Alas! What chance had she (have her prototypes) in this wicked as well as weary world? Of such as these I can but endorse the words said to me a few nights since about a beautiful, homeless, working girl of a very lowly station in life. Alas! poor child, God help her. Ah! truly, for to such as these God's gift and blessing is turned by circumstances into a veritable curse.

Love all mysterious, perplexing indeed is thy influence; the woman who confessed for love's sake, for love's (so called) sake calmly, unflinchingly, murders the husband she has married to save her good name, but whom she has deceived, disliked, scorned all the while. This time she, maybe, remembering a former half-committed murder, leaves woman's favourite method—poison—alone, and follows a surer, less retraceable path, only to prove, as driven by the haunting look on the dead face, she turns accursed away from the possession she has sinned to obtain, and rushes into the terrible darkness of a starless night, the truth of the old adage that "murder will out."

That beheaded head erstwhile stuck up on the railings of old Cripplegate should be a warning to all would-be murderesses, to all sinned against, sinning Catherines of this nineteenth century.

Catherine: A Story of the Reign of Anne. By W. Makepeace Thackeray.

